# Synthesis and Properties of Polyvinylidene Fluoride-Hexafluoropropylene Copolymer/Li_6_PS_5_Cl Gel Composite Electrolyte for Lithium Solid-State Batteries

**DOI:** 10.3390/gels10030199

**Published:** 2024-03-14

**Authors:** Xinghua Liang, Xueli Shi, Lingxiao Lan, Yunmei Qing, Bing Zhang, Zhijie Fang, Yujiang Wang

**Affiliations:** 1 Guangxi Key Laboratory of Automobile Components and Vehicle Technology, Guangxi Transportation Industry Key Laboratory of Vehicle-Road-Cloud Integrated Cooperation, Guangxi University of Science & Technology, Liuzhou 545006, China; lxh304@126.com (X.L.); s1659186234@163.com (X.S.); llx2685062@163.com (L.L.); 18068533778@163.com (Z.F.); 13152528815@163.com (Y.W.); 2 Liuzhou Wuling Automobile Industry Co., Ltd., Liuzhou 545006, China

**Keywords:** gel composite electrolytes, Li_6_PS_5_Cl, ionic conductivity, solid-state batteries

## Abstract

Gel electrolytes for lithium-ion batteries continue to replace the organic liquid electrolytes in conventional batteries due to their advantages of being less prone to leakage and non-explosive and possessing a high modulus of elasticity. However, the development of gel electrolytes has been hindered by their generally low ionic conductivity at room temperature and high interfacial impedance with electrodes. In this paper, a poly (vinylidene fluoride)-hexafluoropropylene copolymer (PVdF-HFP) with a flexible structure, Li_6_PS_5_Cl (LPSCl) powder of the sulfur–silver–germanium ore type, and lithium perchlorate salt (LiClO_4_) were prepared into sulfide gel composite electrolyte films (GCEs) via a thermosetting process. The experimental results showed that the gel composite electrolyte with 1% LPSCl in the PVdF-HFP matrix exhibited an ionic conductivity as high as 1.27 × 10^−3^ S·cm^−1^ at 25 °C and a lithium ion transference number of 0.63. The assembled LiFePO_4_||GCEs||Li batteries have excellent rate (130 mAh·g^−1^ at 1 C and 54 mAh·g^−1^ at 5 C) and cycling (capacity retention was 93% after 100 cycles at 0.1 C and 80% after 150 cycles at 0.2 C) performance. This work provides new methods and strategies for the design and fabrication of solid-state batteries with high ionic conductivity and high specific energy.

## 1. Introduction

Lithium-ion batteries (LIBs) are important components of various devices such as electric vehicles, smart devices, flexible devices, and energy storage systems. Unfortunately, the organic liquid electrolytes currently used in commercial LIBs are problematic due to the risk of leakage and flammability, which could lead to a huge disaster [1,2], and the repeated stripping and plating of rechargeable Li-ion batteries in organic liquid electrolytes produces lithium dendrites, which penetrate the diaphragm and lead to internal short circuits [3]. In order to solve this safety problem and improve the stability of lithium-ion batteries, solid-state batteries based on gel electrolytes have been developed [4], and the problem of dendrites can be solved by replacing the liquid electrolyte with gel electrolytes. A gel electrolyte is an intermediate state between liquid electrolyte and solid electrolyte formed by the combination of liquid electrolyte and solid electrolyte, which can combine the advantages of both, which is a hot spot in today’s research [5].

The PVdF is a semi-crystalline polymer obtained via the polymerization of the 1,1-difluoroethylene monomer. The polymer has the strong electron-absorbing group -CF_2_ and a high dielectric constant, which is favorable for lithium salt dissociation [6]. At the same time, it is characterized by good mechanical properties, high thermal stability, and excellent film-forming properties. The structural symmetry and regular arrangement of PVdF and its high degree of crystallinity lead to its low ionic conductivity at room temperature. The PVdF-HFP obtained after the introduction of hexafluoropropylene (-HFP) groups increases the amorphous phase region of the polymer, which has the advantages of higher room temperature ionic conductivity, lower glass transition temperature and excellent heat resistance. In addition, Li et al. found that the PVdF-HFP polymer could significantly enhance the hydrolysis resistance of sulfide electrolytes, which was attributed to the fact that the PVdF-HFP attached to the surface of sulfide particles could act as a protective isolation layer between the electrolyte and H_2_O, thus avoiding contact-induced hydrolysis reactions and consequently reducing the sulfide’s self-instability caused by moisture absorption [7].

Gel electrolytes are the core of all solid-state lithium batteries. Currently, gel electrolytes can be categorized into oxide-based or sulfide-based gel electrolytes depending on the filler. For example, Zhang et al. [8] used the sol–gel method to prepare highly stable amorphous LLTO films and used strontium as a dopant to prepare Li_0.35_La_0.5_Sr_0.05_TiO_3_. When the doping amount of strontium is 5%, the ionic conductivity reaches 8.38 × 10^−5^ S cm^−1^ at 30 °C, which is an order of magnitude higher than that of undoped LLTO. Mishra G K et al. [9] prepared PEO/LSiPS (Li_10_SiP_2_S_12_) CGPEM via the solution casting method. The ionic conductivity it can achieve is 7.53 × 10^−4^ S cm^−1^. Among the sulfide-based gel electrolytes, LPSCl has attracted much attention because of its high ionic conductivity and good mechanical deformation ability [10]. Moreover, LPSCl belongs to a kind of active filler, and the addition of a certain amount of active filler can increase the amorphous region of the electrolyte, which helps to improve the excellent performance of the gel electrolyte.

Gel electrolytes can be categorized into two main groups, the polymer gel electrolytes and organic–inorganic gel composite electrolytes. For example, Liang et al. [11] prepared a dense gel film with certain flexibility by blending PVDF and PEO. After absorbing the liquid electrolyte of lithium hexafluorophosphate liquid, it showed excellent performance, and the ionic conductivity reached 0.28 × 10^−3^ S cm^−1^ at a room temperature of 25 °C. Shi et al. [12] used PVDF as a matrix to blend PVDF-HFP, PEO, and PMMA in a certain proportion to obtain a polymer blend film and then absorbed liquid electrolyte to prepare a gel electrolyte. The prepared gel electrolyte had good mechanical properties and porous structure. At room temperature, the ionic conductivity could reach 0.81 × 10^−3^ S cm^−1^, the affinity with the electrode was obviously improved, and the thermal stability was also improved. Zhou et al. [13] successfully prepared the electrolyte film via electrospinning by adding TiO_2_ nanofillers to the gel polymer electrolyte based on PVDF/PMMA. After adding the inorganic nanofillers, the crystallinity of the film was significantly reduced, and the mechanical properties and ionic conductivity were significantly improved. The ionic conductivity at room temperature reached 3.9 × 10^−3^ S cm^−1^, and the electrochemical window reached 5.1 V. Polymer gel electrolytes are made of polar polymers and lithium salt complexes, which have good flexibility, can effectively inhibit the growth of dendrites, and are easy to be produced on a large scale, but the ionic conductivity of the polymer electrolyte is low, and it is easy to be decomposed under high pressure [14]. Although inorganic gel electrolytes have the advantages of good thermal stability and high ionic conductivity, there is a large solid/solid contact resistance between the electrode and the electrolyte, and their high mechanical strength may impede the ionic transport. Therefore, in this work, gel composite electrolytes is selected. Gel composite electrolytes combine the advantages of organic polymers and inorganic gel electrolytes with the advantages of high ionic conductivity, cyclic stability, and good ductility and the long-standing shortcomings of low ionic conductivity, and the gel composite electrolytes are lightweight and flexible like polymers, and they have the strength and stability of inorganic electrolytes [15], so the gel composite electrolytes are a promising alternative.

So far, various types of gel electrolytes have been explored. According to the different fillers, the gel electrolyte can be divided into gel electrolytes containing oxides chalcogenide PeroxidLi_3_xLa_0.67_-xTiO_3_ (LLTO [16]), Garnet (Li_7_La_3_Zr_2_O_12_ (LLZO) [17] and so on, the gel electrolyte containing sulfides glass–ceramic phase (Li_3_PS_4_ [18]), sulfurized crystalline lithium superionic conductors (Thio-LISICON [19]) and sulfur–silver–germanium ore type (Li_10_GeP_2_S_12_(LGPS) [20] and LPSCl [21]). For example, Li et al. [22] constructed a composite film PVdF/LLTO/LiClO_4_ with an ultrathin flexible structure by utilizing a one-dimensional LLTO nanowire with a biomimetic cellular structure, which was incorporated into PVdF/LiClO_4_. The ionic conductivity of this composite film was 5.8 × 10^−4^ S·cm^−1^ at room temperature. Zegeye et al. [23] dispersed zero-dimensional LLZTO powders into an acetonitrile solution of PEO/LiTFSI and designed and prepared an ultrathin PEO/LLZTO/LiTFSI composite electrolyte film in the range of 7~10 μm by spin-coating the electrolyte solution onto the positive electrode sheet or copper sheet. Then, the composite electrolyte film-coated positive electrode and copper sheet were molded together to construct a button cell with an electrolyte film thickness of 15~20 μm. The test results showed that the ionic conductivity of the composite electrolyte film was 4.76 × 10^−4^ S·cm^−1^ at room temperature. Fan et al. [24] investigated the preparation of a PEO/LLZTO/LiTFSI composite electrolyte with 10% (mass fraction) of LLZTO by adding the nanofiber LLZTO to PEO/LiTFSI. The test results showed that the ionic conductivity of the designed composite electrolyte was 2.13 × 10^−4^ S·cm^−1^ at room temperature. Zhao [25] et al. composited Li_10_GeP_2_S_12_ (LGPS) particles as active fillers with a PEO-LiTFSI electrolyte and investigated the effect of the LGPS additions on the ionic conductivity of the polymer system. The comparative the results are shown in Table 1, which indicate that the optimal addition content of LGPS is 1 wt%, and the highest ionic conductivity is 1.2 × 10^−3^ S·cm^−1^. It can be seen that the generally low ionic conductivity of oxide gel composite electrolytes hinders the future development of solid-state batteries. Compared with oxides, the radius of sulfur ions is larger than that of cations, the electronegativity is smaller, and the binding of lithium ions by sulfide gel electrolytes is lower compared to oxide gel electrolytes [26], which makes the ion transport channel formed by sulfide become wider than that of oxide electrolytes, and it is easier to transport lithium ions, thus showing higher ionic conductivity, which can effectively solve the lower ionic conductivity of gel electrolytes [27].

In this work, we combine polymer PVdF-HFP and sulfide LPSCl powder with an appropriate amount of LiClO_4_ stirred well to obtain a sulfide gel composite electrolyte film via thermosetting. The gel electrolyte film was assembled with a lithium metal anode to form an LiFePO₄//gel composite electrolyte film||Li solid-state battery. This solid-state battery exhibits good electrochemical performance. This work provides feasible strategies and methods for realizing gel composite electrolytes with high ionic conductivity and high mechanical properties and the development of solid-state batteries, and it lays a good foundation for the research of gel composite electrolytes and the development of solid-state batteries.

## 2. Results and Discussion

**Figure 1:**
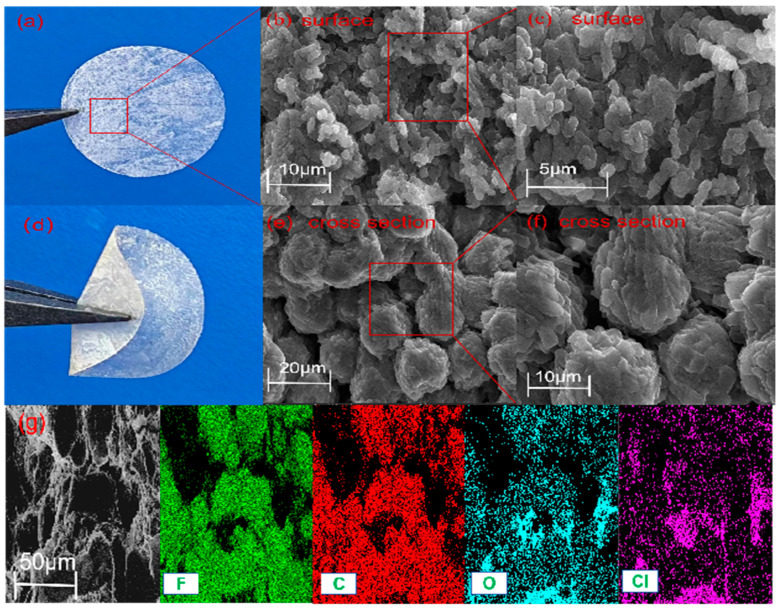
(**a**,**d**) Macroscopic picture of PVdF-HFP/LiClO_4_ gel electrolyte film. (**b**) Moreover, 10 μm SEM image of the surface morphology of PVdF-HFP/LiClO_4_. (**c**) Furthermore, 5 μm SEM image of the surface morphology of PVdF-HFP/LiClO_4_. (**e**) Moreover, 20 μm SEM image of the cross-section morphology of PVdF-HFP/LiClO_4_. (**f**) Furthermore, 10 μm SEM image of the cross-section morphology of PVdF-HFP/LiClO_4_. (**g**) Moreover, 50 μm SEM image and EDS mappings of PVdF-HFP/LiClO_4_.

The morphology of the gel electrolyte film of PVdF-HFP/LiClO_4_ is shown in Figure 1a,d, from which it can be seen that the surface of the film is uniform and textured, flexible and plastic. Figure 1b,c show the SEM images of the gel electrolyte film of PVdF-HFP/LiClO_4_. It can be seen that the surface of the film has the granularity of lithium perchlorate salt and has a uniform pore appearance, but the matrix phase is relatively more continuous. As can be seen in Figure 1e,f, after doping the PVdF-HFP with lithium perchlorate salt, the particles of lithium perchlorate salts, which form interconnected clusters, form fast Li^+^ permeation channels that aid in the propagation of lithium ions [28]. Figure 1g shows the energy-dispersive spectroscopy (EDS) mapping images of F, C, O, and Cl of 0% LPSCl, which further proves the uniform distribution of each element in the GCEs.

**Figure 2:**
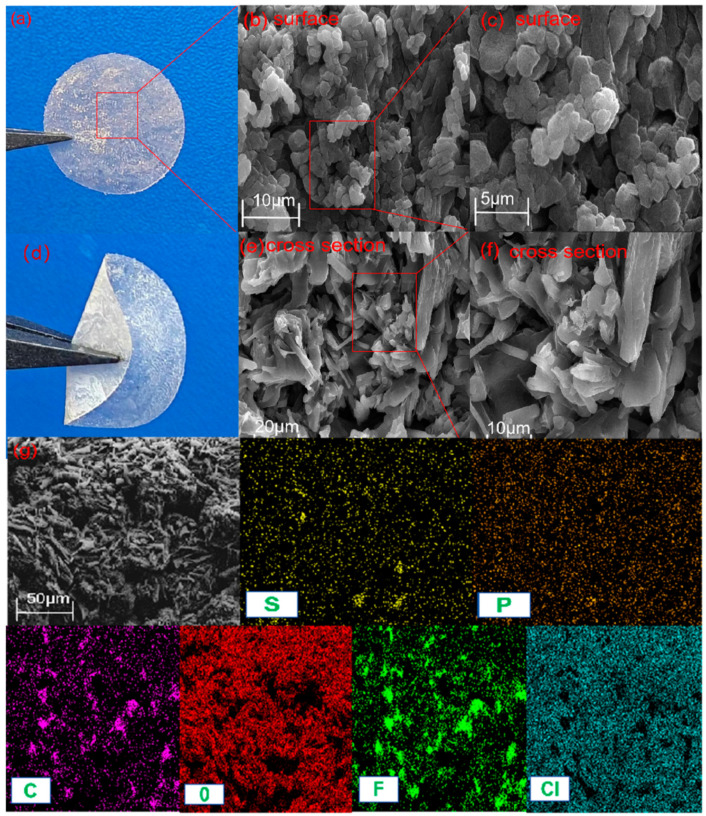
(**a**,**d**) Macroscopic picture of PVdF-HFP/LPSCl/LiClO_4_ gel composite electrolyte film. (**b**) Moreover, 10 μm SEM images of the surface morphology of PVdF-HFP/LPSCl/LiClO_4_ gel composite electrolyte film. (**c**) Furthermore, 5 μm SEM images of the surface morphology of PVdF-HFP/LPSCl/LiClO_4_ gel composite electrolyte film. (**e**) Moreover, 20 μm SEM images of the cross-sectional morphology of PVdF-HFP/LPSCl/LiClO_4_ gel composite electrolyte film. (**f**) Furthermore, 10 μm SEM images of the cross-sectional morphology of PVdF-HFP/LPSCl/LiClO_4_ gel composite electrolyte film. (**g**) Moreover, 50 μm SEM image and EDS mappings of PVdF-HFP/LiClO_4_/LPSCl distributions in the SCE membrane.

Figure 2a,d show the morphology of the gel electrolyte film formed by adding 1% LPSCl, from which it can be seen that this film shows a light yellow color and the film can withstand deformations such as folding and winding, which proves that it is robust, and the soft nature of the gel composite electrolyte film can also improve its good contact with the electrodes.

Figure 2c,d show the surface morphology of the PVdF-HFP/LPSCl/LiClO_4_ gel composite electrolyte film formed by the addition of 1% LPSCl. From the cross-section of Figure 2e,f, it can be clearly seen that after the addition of a substance like LPSCl, the PVdF-HFP/LiClO_4_ changes from a dispersed salt cluster to an interconnected phase, and many long fibrous strips [29] appear tightly interwoven with each other to form a continuous and dense electrolyte film, and the presence of tight channels in it helps the ionic transport and contributes to the improvement of the ionic conductivity of the gel electrolyte. Figure 2g shows the energy-dispersive spectroscopy (EDS) mapping images of F, C, O, and Cl of 1% LPSCl, which further proves the uniform distribution of LPSCl in the GCEs.

**Figure 3:**
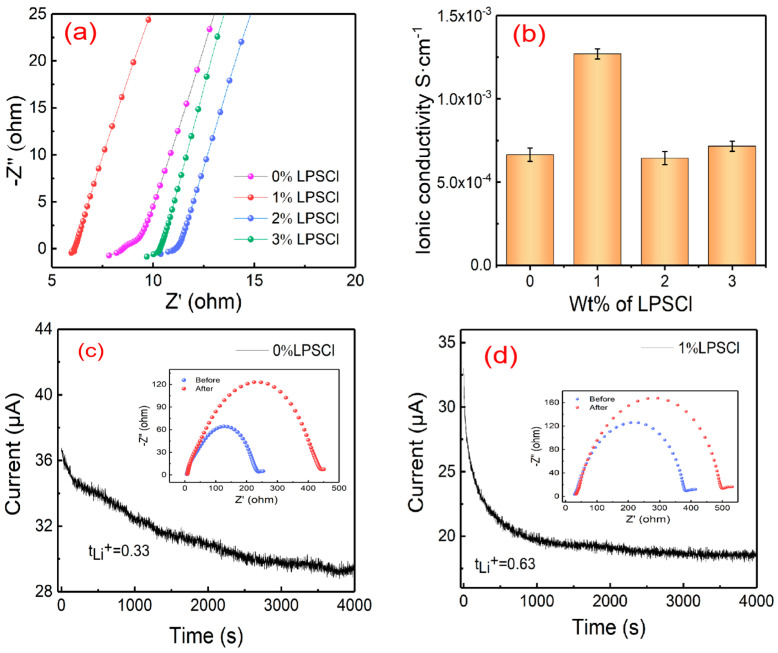
(**a**) Impedance values of the battery electrolyte corresponding to the addition of different proportions of LPSCl for PVdF-HFP/LPSCl/LiClO_4_. (**b**) Ionic conductivity of the gel composite electrolyte film corresponding to the addition of different proportions of LPSCl. (**c**) Lithium-ion transference number of PVdF-HFP/LiClO_4_ gel composite electrolyte. (**d**) Lithium-ion transference number of PVdF-HFP/LPSCl/LiClO_4_ gel composite electrolyte.

In order to investigate the effect of different LPSCl mass fractions on gel composite electrolyte films. The impedance values of the gel electrolytes were determined at 0% wt, 1% wt, 2% wt, and 3% wt mass ratios of LPSCl to the polymer matrix PVdF-HFP, respectively. Figure 3a shows the impedance value of the gel composite electrolyte, and Figure 3b shows the trend of ionic conductivity with different amounts of LPSCl added, and from the figure it can be seen that 1% wt has the highest ionic conductivity. From the measured impedance value of the electrolyte and the calculation of Equation (1), it can be found that the ionic conductivity of the gel composite electrolyte film shows a trend of increasing and then decreasing with the increase in the amount of LPSCl added, and the ionic conductivity values are, respectively, 6.55 × 10^−4^ (L_1_ = 112 μm), 1.27 × 10^−3^ (L_2_ = 156 μm), 6.44 × 10^−4^ (L_3_ = 144 μm), and 7.16 × 10^−4^ (L_4_ = 149 μm). Since the ionic conductivity in the sulfide-based gel composite electrolyte is mainly determined via Li^+^ migration between LPSCl particles [30], the number of carriers Li^+^ in LPSCl complexed with C and F protons in the PVdF-HFP matrix increases as the concentration of Li^+^ rises with the increase in LPSCl content. The ionic conductivity of the gel composite electrolyte increases consequently, and when the concentration of LPSCl reaches 3% wt, LPSCl has formed saturation in the system, and the carriers in the excess LPSCl cannot be completely dissolved but gather together in the form of ionic clusters [31], forming certain agglomerations and precipitations. Losing a part of the carriers in the LPSCl and reducing the number of available carriers leads to the impedance of the transport, which affects the ionic conductivity of the gel composite electrolyte, and from Figure 3a, the resistance of the gel composite electrolyte is the smallest when the proportion of LPSCl added is 1%, which is 6.113 Ω. From Figure 3b, the ionic conductivity of 1% wt is up to 1.27 × 10^−3^ S·cm^−1^. Compared to the ionic conductivity of the gel electrolyte without the addition of LPSCl (6.55 × 10^−4^ S·cm^−1^), the increase in ionized Li^+^ of the gel composite electrolyte with the addition of LPSCl is associated with an increase in the carrier concentration of Li^+^ [32], and as can be seen from the cross-sectional views in Figure 1e,f and Figure 2e,f, after the addition of LPSCl, the morphological characteristics of the film changed from clusters to striped fibers intertwined together. Not only is there an increase in lithium ion channels, but this structure is more conducive to lithium ion transport, which explains the higher lithium ion migration number of 1% LPSCl (*t_Li_*_+_ = 0.63) than that of 0% LPSCl (*t_Li_*_+_ = 0.33) (Figure 3c,d). Therefore, the research in this paper will test and study the performance of the gel composite electrolyte film as well as the assembled solid-state battery on the basis of the 1% ratio. Figure 3c shows the lithium-ion transference number of the PVdF-HFP/LiClO_4_ gel composite electrolyte, which is calculated to be 0.33 after Equation (2), and Figure 3d shows the lithium-ion transference number of the PVdF-HFP/LPSCl/LiClO_4_ gel composite electrolyte is 0.63.

**Figure 4:**
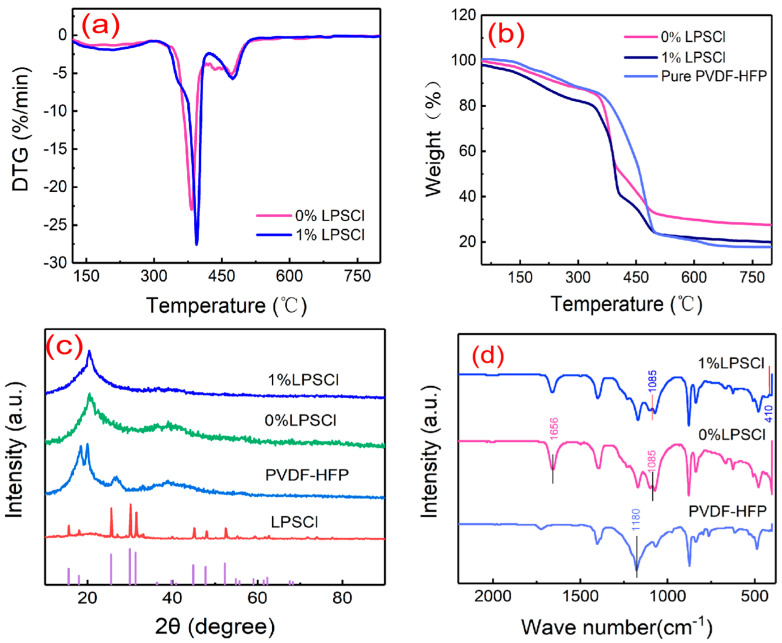
(**a**) DTG results of PVdF-HFP/LiClO_4_ gel electrolyte film and PVdF-HFP/LPSCl/LiClO4 gel composite electrolyte film; (**b**) thermogravimetric analysis curves of PVdF-HFP, PVdF-HFP/LiClO_4_, and PVdF-HFP/LPSCl/LiClO_4_ gel composite electrolyte films; (**c**) X-ray diffraction (XRD) patterns of PVdF-HFP, PVdF-HFP/LiClO_4_ gel electrolyte film, and PVdF-HFP/LPSCl/LiClO_4_ gel composite electrolyte film; (**d**) Fourier-transform infrared (FTIR) spectral curves of PVdF-HFP, PVdF-HFP/LiClO_4_ gel electrolyte film, and PVdF-HFP/LPSCl/LiClO_4_ gel composite electrolyte film.

In order to explore the thermal stability of the gel electrolyte films, they were tested and analyzed using a heat loss analyzer, and Figure 4a,b show the DTG and TGA analysis of PVdF-HFP, PVdF-HFP/LiClO_4_, and PVdF-HFP/LPSCl/LiClO_4_ films. From Figure 4a, it can be observed that the two proportions of membranes, 0% LPSCl and 1% LPSCl, show similar thermogravimetric behaviors, but the highest pyrolysis temperature reached for the first time in 0% LPSCl proportions membranes is 382 °C, while the highest pyrolysis temperature reached for the first time in 1% LPSCl proportions membranes is 395 °C. From the Figure 4b, it can be seen that the weight loss of the PVdF-HFP, PVdF-HFP/LiClO_4_, and PVdF-HFP/LPSCl/LiClO_4_ films is almost negligible before 150 °C. A huge weight loss occurs at about 360 °C for PVdF-HFP, indicating that PVdF-HFP decomposes at about 360 °C. At about 175 °C, trace losses of 1% LPSCl occur due to the evaporation of trace amounts of residual DMF and water vapor in the gel electrolyte film. At about 330 °C, weight losses occur in both the 0% LPSCl and 1% LPSCl gel electrolyte films; this is due to the fact that LiClO_4_ in PVdF-HFP/LiClO_4_ and PVdF-HFP/LPSCl/LiClO_4_ reduces the crystallinity of the films and improves the amorphous region of the gel composite electrolyte films so that the decomposition temperatures of the gel electrolyte films are reduced in comparison with those of PVdF-HFP [33]. Through Figure 4a,b, it can be seen that the decomposition temperature of the 1% LPSCl film was higher than that of the 0% LPSCl film after the addition of 1% LPSCl, which proved that LPSCl helped to improve the thermal stability of the gel composite electrolyte film.

Figure 4c shows the measured X-ray diffraction (XRD) patterns of PVdF-HFP, LPSCl, PVdF-HFP/LiClO_4_ (0% LPSCl) gel films, and PVdF-HFP/LPSCl/LiClO_4_ (1% LPSCl) gel composite electrolyte films. As can be seen from the figures, around 18°, 20°, 26°, and 39° are the crystal structures of the PVdF-HFP-based gel electrolyte. The diffraction peak positions of the LPSCl samples were basically consistent with the standard card, corresponding to the composition of the same substance as LPSCl [34], and no obvious LPSCl peaks were observed in the formed gel electrolyte film, suggesting that the LPSCl was completely dissolved in the polymer. After the incorporation of lithium perchlorate salt in PVdF-HFP, the 0% LPSCl gel electrolyte film showed peaks only around 2θ = 20° and 2θ = 39°. After the introduction of LPSCl as a sulfide on this basis, the significant decrease in the intensity of the diffraction peaks disappeared around 2θ = 39°, which indicates that the introduction of LPSCl powder significantly reduces the crystallinity of PVdF-HFP and leads to more amorphous regions, which contributes to ionic conduction in gel composite electrolyte films [35].

The Fourier-transform infrared (FT-IR) spectra of PVdF-HFP, PVdF-HFP/LiClO_4_, and PVdF-HFP/LPSCl/LiClO_4_ are given in Figure 4d, and the performance of the films with two proportions of 0% LPSCl and 1% LPSCl is compared. As can be seen in the FT-IR spectrum in Figure 4d, there is a strong peak at 1180 cm^−1^ in PVdF-HFP, which is the stretching vibration of CH_2_ and CH_3_ groups in PVdF-HFP, and there is a characteristic peak belonging to LiClO_4_ at 1656 cm^−1^ in 0% LPSCl. An interaction between dissociated Li^+^ and -CF_2_ was present in PVdF-HFP at 410 cm^−1^ in 1% LPSCl, and the characteristic peak at 1085 cm^−1^ is LiCl dissociated in the electrolyte [36].

**Figure 5:**
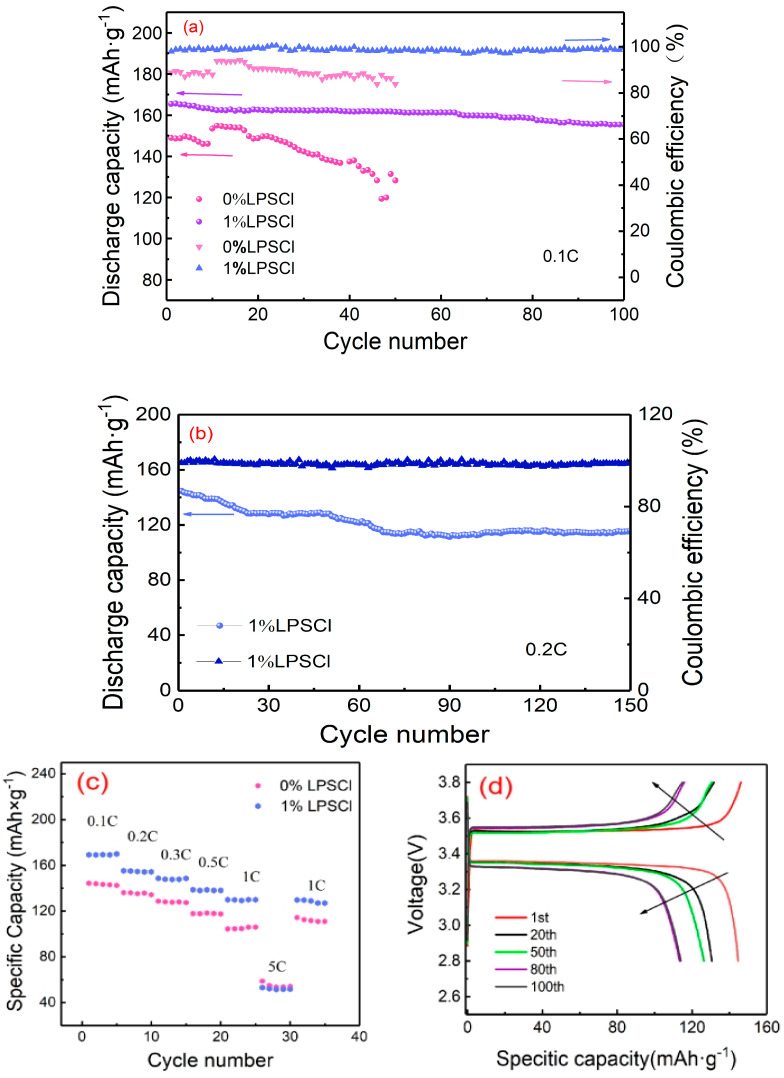
(**a**) Comparative cycling performance graphs of PVdF-HFP/LiClO_4_ and PVdF-HFP/LPSCl/LiClO_4_ solid-state batteries at 0.1 C; (**b**) graphs of current cycling performance of PVdF-HFP/LPSCl/LiClO_4_ solid-state batteries at 0.2 C; (**c**) graphs of PVdF-HFP/LiClO_4_ and PVdF-HFP/LPSCl/LiClO_4_ solid-state battery performance from 0.1 C to 5 C multiplication rate; (**d**) charge–discharge curve of PVdF-HFP/LPSCl/LiCLlO_4_ solid-state battery at 0.2 C.

Figure 5 shows the plots of multiplicity comparisons, current cycling performance at 0.1 C and 0.2 C, and charging/discharging specific capacity curves for different number of turns at 0.2 C for two proportions of solid-state batteries with 0% LPSCl and 1% LPSCl. From Figure 5a, it can be seen that the cycling curve of 0% LPSCl is extremely unstable, and this instability is attributed to the high crystallinity of PVdF-HFP/LPSCl, while the addition of LPSCl reduces the crystallinity of the film, which facilitates the transport of lithium ions [37]. The cycling curves stabilized with the addition of 1% LPSCl and the capacity retention of the cells reached 93% after 100 cycles. Figure 5b shows the current cycling curve of 1% LPSCl at 0.2 C, from which it can be seen that the cycling is stable, the coulombic efficiency is maintained at about 92%, and the capacity retention is 80% after 120 cycles. Figure 5c shows the comparison of the solid-state cell with two proportions of 0% LPSCl and 1% LPSCl at different multiplications, from which it can be seen that after the addition of LPSCl to the PVdF-HFP/LiClO_4_, the rate performance is improved. It can be seen from the figure that this enhancement is attributed to the superior ion transport path and the uniform distribution of dense fiber strips and tight holes in the composite solid electrolyte after the addition of LPSCl, which is conducive to the transport and stability of lithium ions. Therefore, the battery assembled with this solid electrolyte film has a specific discharge capacity of 169 mAh·g^−1^ at 0.1 C. Figure 5d shows the constant current charging/discharging curves of a 1% LPSCl solid-state battery at 0.2 C and 25 °C. It can be seen from the figure that the capacity and voltage plateau remain stable, which is due to the compactness of the membrane after adding 1% LPSCl, which is beneficial for the stability of ion transport.

**Figure 6:**
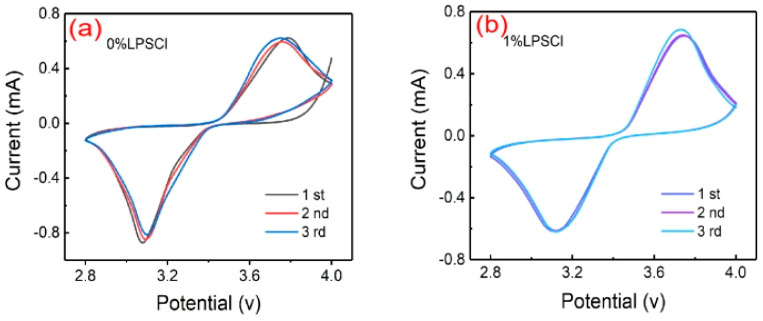
(**a**) CV curve of PVdF-HFP/LiClO_4_ solid-state battery; (**b**) CV curve of PVdF-HFP/LPSCl/LiClO_4_ solid-state battery.

Figure 6 shows the electrochemical window curves obtained using cyclic voltammetry for 0% LPSCl and 1% LPSCl. A comparison reveals that the oxidation and reduction peaks of the 1% LPSCl solid-state battery are highly symmetric. Additionally, three full curves of 1% LPSCl coincide in height, proving that the reaction between the active substance and the electrode is reversible, demonstrating a certain stability of the battery and enhanced conductivity, which is also related to the film’s structure. In comparison, the CV curve of 0% LPSCl is less smooth and less stable. Thus, the addition of LPSCl reduces the polarization degree of the battery, but the reduction peaks of the 0% sample are higher than the 1% sample, which means that the electrode reaction of the 0% sample is faster and the reaction kinetics are faster, but it does not mean that it has a better electrochemical performance [38]. In addition, we observed that 1% LPSCl has a higher three-turn overlap and good symmetry of the redox peaks, which suggests that the reaction is more reversible and the electrolyte material has good electrochemical performance, which is also confirmed by the electrochemical performance data in Figure 5a–c.

## 3. Conclusions

In this experiment, polymer PVdF-HFP and sulfide LPSCl were stirred together, gel composite electrolyte films were prepared via the process of thermosetting, and the performance of a solid-state battery was measured by measuring and comparing the performance of the film with and without adding 1% LPSCl and after assembling the gel composite electrolyte film into a solid-state battery. The conclusions are as follows:

(1) The gel electrolyte films of 0% LPSCl and 1% LPSCl are both flexible, but the internal structure of the 1% LPSCl gel electrolyte film is more pyknotic, presenting a fibrous dense-type structure of the film to help lithium-ion transport. The ionic conductivity of the gel electrolyte film of 0% LPSCl is 6.55 × 10^−4^ S·cm^−1^, and the lithium-ion mobility number is 0.33; The ionic conductivity of the gel electrolyte film with 1% LPSCl reached 1.27 × 10^−3^ S·cm^−1^, and the lithium-ion transference number reached 0.63; Moreover, the gel electrolyte film with 1% LPSCl did not decompose until 395 °C, and it had extremely high thermal stability.
(2) The gel electrolyte film with 1% LPSCl exhibited a higher rate performance compared to the gel electrolyte film with 0% LPSCl, which had a discharge specific capacity of 130 mAh·g^−1^ at 1 C and 54 mAh·g^−1^ at 5 C.
(3) The 1% LPSCl gel electrolyte film has a more stable cycling performance than the 0% LPSCl gel electrolyte film. The 0% LPSCl gel electrolyte film is extremely unstable in cycling at 0.1 C, whereas the 1% LPSCl gel electrolyte film has a first-turn discharge specific capacity of 165 mAh·g^−1^ in cycling at 0.1 C and still has a 93% capacity retention rate in cycling after 100 turns. The 1% LPSCl gel electrolyte film has a first turn discharge capacity of 139 mAh·g^−1^ at 0.2 C and still has 80% capacity retention after 150 turns, which proves that the addition of LPSCl is more helpful for the future development of gel electrolytes and solid-state batteries. This experimental program provides new strategies and methods for the development of gel composite electrolytes and solid-state batteries with high ionic conductivity.

## 4. Materials and Methods

### 4.1. Materials

*N*,*N*-dimethylformamide (DMF, Macklin, Shanghai, China), polyethylene (vinylidene fluoride-hexafluoropropylene) (PVdF-HFP, Mn = 600,000, Arkema, Colombe, France), lithium perchlorate salt (LiCLO_4_, Aladdin, Shanghai, China)), and lithium phosphorus sulfur chloride (LPSCl, Kejing Star, Shenzhen, China) powders.

### 4.2. Preparation of Gel Composite Electrolytic Film

**Figure 7:**
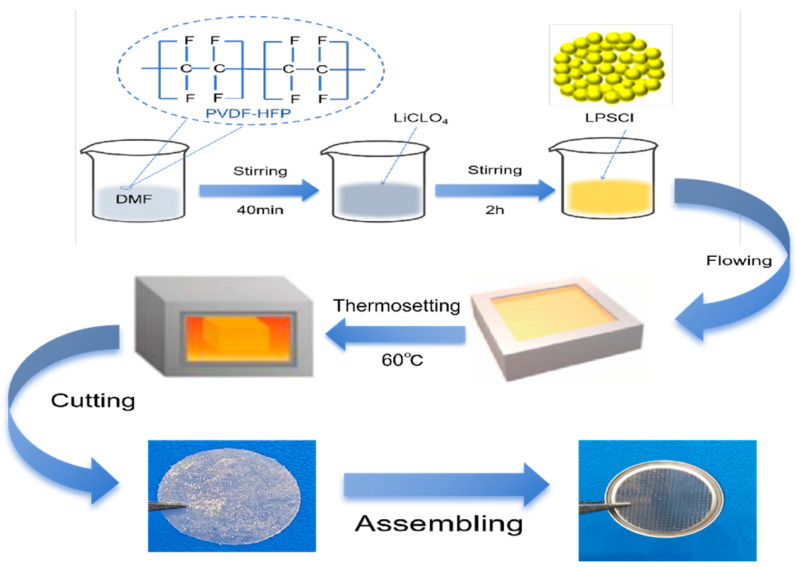
Flowchart of gel composite electrolyte film preparation.

The gel composite electrolytic film containing PVdF-HFP and LPSCl was prepared via the thermosetting method, and the synthesis process is shown in Figure 7. Moreover, 2 g of PVdF-HFP was dissolved in 8 g of DMF and stirred at 55 °C for 40 min. After the solution became a transparent mucus, 0.5 g of LiClO_4_ was added and stirred for 2 h to present a homogeneous slurry, then LPSCl powder (later called 0% LPSCl and 1% LPSCl), which accounted for 0%, 1%, 2%, and 3% of the mass of the PVdF-HFP, was added to the solution separately and stirred for 5 h, and the stirred mucus was first poured onto a tetrafluoroethylene plate via the casting method and then formed into a sulfide composite gel electrolyte film via the thermosetting method at 60 °C.

### 4.3. Preparation of Positive Electrodes

Under normal air conditions, firstly, all the required drugs were dried in a drying oven, and 1.6 g of lithium iron phosphate (LiFeO_4_), 0.2 g of conductive carbon black, and 0.2 g of poly(vinylidene fluoride) (PVdF) were weighed for spare use. After putting the rotor into a clean and dry beaker, a small amount of *N*-methyl pyrrolidone (NMP) reagent (the amount of reagent can be enough to moisten the wall) and PVdF were put into the beaker in turn, and the sample was magnetically stirred for 1.5 h. At the same time, lithium iron phosphate and conductive carbon black were poured into an onyx mortar, fully ground for 40 min, and then poured into the beaker that was being magnetically stirred, and the beaker that was being stirred was covered with a plastic wrap under air conditions to prevent the volatilization of the materials. After 4 h of magnetic stirring, a uniform slurry was obtained; the slurry was uniformly poured onto the aluminum foil paper, and the slurry was uniformly coated with a thickness of 30 μm by a coating machine, and the coated aluminum foil paper was dried in a drying oven for 36 h~48 h. The dried electrode sheet was pressed into an electrode sheet with a diameter of 16 mm in a 10 MPa platen press for spare use.

### 4.4. Composite Solid-State Battery Assembly

Assemble the CR2025 button cell in the glove box filled with argon gas. Before assembling the battery, the GCEs were immersed in organic liquid electrolyte (1 mol L^−1^, LiPF_6_ in EC:DMC:EMC = 1:1:1 Vol% with 1% VC) for 20 min in an argon-filled glove box. Then, the prepared gel composite electrolyte film in accordance with the order from top to bottom for the negative shell/lithium plate/composite gel electrolyte film/positive/positive shell was assembled in the order of what was spare.

### 4.5. Characterization Measurements

The micromorphology of the gel composite electrolyte was observed via scanning electron microscopy (SEM; Gemini, SEM 500, ZEISS Inc., Jena, Germany). X-ray diffraction (XRD, D8-ADVANCE, Bruker, Karlsruhe, Germany, Cu, 40 kV, 40 mA) was used for the physical analysis of the test material, and the radiation source was Cu-Ka rays at a radiation angle between 10° and 90° in steps of 0.1°, a tube voltage and tube current of 40 kV and 30 mA, respectively, and a scanning speed of 0.10°·min^−1^ to analyze the physical phase of the samples. The weight of the test samples was changed during the temperature ramp-up or ramp-down. The changes in reaction kinetics and the thermal and oxidative stability of the materials in different atmospheres can be probed using thermal weight loss analysis (TG).

### 4.6. Electrochemical Measurements

Under normal operating conditions, ionic conductivity is an important index parameter for judging the migration ability of all ions as well as whether the film can be used to assemble the battery and put it into use. The electrochemical impedance spectroscopy (EIS) was measured on the electrochemical workstation of the DH7000, the tailored film was assembled into a steel sheet/film/steel sheet half-buckle cell, and the measured voltage amplitude was set to 10 mV and the frequency to 0.1 Hz~1.0 MHz. The cut film was assembled into a steel/film/steel half-button cell, the amplitude of the measured voltage was set to 10 mV, the frequency was 0.1 Hz to 1.0 MHz, and the ionic conductivity at two ratios was calculated according to Equation (1) to analyze the energy utilization.

(1) $$\sigma =L\frac{R}{S}$$

where *σ* denotes the ionic conductivity, *L* is the thickness of the electrolyte, *S* denotes the contact area between the electrolyte and the test electrode, and *R* is the impedance value of the cell electrolyte measured by EIS.

The lithium-ion transference number was tested on the electrochemical workstation of the DH7000, and the lithium symmetric battery was tested at 0.5 mV for a duration of 4000 s. The lithium-ion transference number of the gel electrolyte was obtained by using Equation (2) (*t_Li_*_+_).

(2) $$t_{{Li}^{+}}=\frac{I_{s}(∆V-I_{0}R_{0})}{I_{0}(∆V-I_{s}R_{s})}$$

*I*_0_ and *I_s_* are the current values after DC polarization has started and stabilized, *R*_0_ and *R_s_* are the impedance values before and after polarization, and ∆*V* is the voltage value applied to the cell terminals.

The cyclic voltammetry (CV) test was performed on a DH700 electrochemical workstation with a scan rate of 0.1 mV~1 mV. By assembling the gel composite electrolyte film into LPF/LPSCl film/Li (10^6^ Hz–0.01 Hz, 10 mV, AC amplitude value), the voltage range of the debugging test system was 2.5 V~4.2 V, and the scan rate was 5 mV/s. The current and voltage variations were observed. The voltage changes were observed to determine whether the cell electrode was reversible or not.

## Figures and Tables

**Table 1:** Comparison of ionic conductivity of different types of gel composite electrolyte films.

Organic Polymer Substrate	Type of Filler	Lithium Salt Type	Ionic Conductivity	Reference
PVdF	LLZTO	LiClO_4_	5.8 × 10^−4^ S·cm^−1^	[16]
PEO	LLZTO	LiClO_4_	4.76 × 10^−4^ S·cm^−1^	[17]
PEO	LLZTO	LiTFSI	2.13 × 10^−4^ S·cm^−1^	[18]
PEO	LGPS	LiTFSI	1.2 × 10^−3^ S·cm^−1^	[19]

## Data Availability

Data are contained within the article.
